# The Group of Treatment Resistant Schizophrenias. Heterogeneity in Treatment Resistant Schizophrenia (TRS)

**DOI:** 10.3389/fpsyt.2018.00757

**Published:** 2019-01-30

**Authors:** Bruce J. Kinon

**Affiliations:** Lundbeck North America, Deerfield, IL, United States

**Keywords:** schizophrenia, antipsychotic drug, treatment resistant, clozapine, dopamine, first-episode schizophrenia (FES), magnetic resonance spectroscopy (MRS), positron emission tomography–PET

## Abstract

Schizophrenia is composed of a heterogeneous group of patient segments. Our current notion of the heterogeneity in schizophrenia is based on patients presenting with diverse disease symptom phenotypes, risk factors, structural and functional neuropathology, and a mixed range of expressed response to treatment. It is important for clinicians to recognize the various clinical presentations of resistance to treatment in schizophrenia and to understand how heterogeneity across treatment resistant patient segments may potentially inform new strategies for the development of effective treatments for Treatment Resistant Schizophrenia (TRS). The heterogeneity of schizophrenia may be reduced by parsing patient segments based on whether patients demonstrate an adequate or inadequate response to treatment. In our current concept of TRS, TRS is defined as non-response to at least two adequate trials of antipsychotic medication and is estimated to affect about 30% of all patients with schizophrenia. In this narrative review, the author discusses that the demonstration of inadequate response to antipsychotic drugs (APDs) may infer that some TRS patients may be suffering from a non-dopamine pathophysiology since D2 receptor antagonist-based treatment is ineffective. Preliminary neurobiological findings may further support the pathophysiologic distinction of TRS from that of general schizophrenia. Investigation of the basis for heterogeneity in TRS through the systematic investigation of relevant “clusters” of similarly at risk individuals may hopefully bring us closer to realize a precision medicine approach for developing effective therapies for TRS patient segments.

Schizophrenia is composed of a heterogeneous group of patient segments. This heterogeneity has been long recognized. In Bleuler's treatise on schizophrenia, *The Group of Schizophrenias*, he writes of the heterogeneity of symptoms, for example primary, secondary or accessory, as well as of the heterogeneity of outcomes; good, fair, and poor (1). Our current notion of the heterogeneity in schizophrenia is similarly based on patients presenting with diverse phenotypes characterized by differing symptoms and signs of illness as well as life course, multiple risk factors leading to disease including a complex genetic loading, a broad spectrum of neurobiological features suggesting a pathophysiology of structure and function that is not necessarily shared by all patients, and a mixed range of expressed response to treatment.

Heterogeneity in response to antipsychotic drug (APD) treatment is seen across the course of schizophrenia. Some patient segments demonstrate APD responsiveness throughout their illness, others demonstrate resistance to treatment only after many years, or only a few years, of treatment responsiveness. Others still may be found to respond poorly to APD treatment since their first episode of psychosis (Figure 1). It is important for clinicians to recognize the various clinical presentations of resistance to treatment in schizophrenia and to understand how heterogeneity across treatment resistant patient segments may potentially inform new strategies for the development of effective treatments for TRS. In addition, it is crucial for clinicians to rule-out “pseudo-TRS” due to inadequacy of APD exposure from either poor adherence (2), under-dosing, ultrarapid drug metabolism (3), or limited length of treatment duration (4).

**Figure 1:**
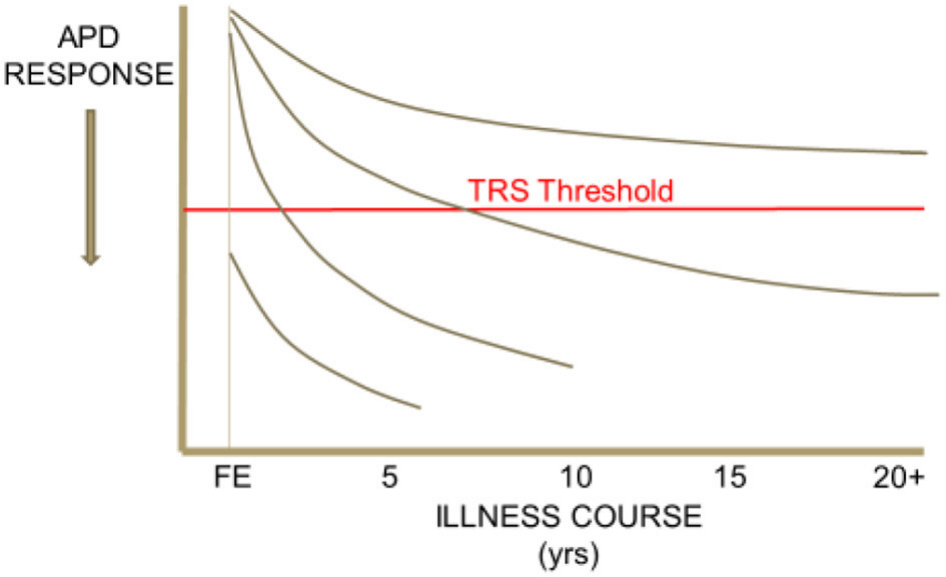
Heterogeneity in the trajectory of response to APD treatment over the illness course of schizophrenia. This schematic drawing illustrates that some patient segments may demonstrate APD responsiveness throughout their illness, others demonstrate resistance to treatment only after an initial period of treatment responsiveness, and others still may be found to respond poorly to APD treatment since their first episode of psychosis.

The heterogeneity of schizophrenia may be reduced by bifurcating patient segments based on whether patients demonstrate an adequate or inadequate response to treatment. In our current concept of TRS, TRS is defined as non-response to at least two adequate trials of antipsychotic medication (4). TRS is estimated to affect about 30% of all patients with schizophrenia (5). As presently defined, TRS reflects the persistence of prominent positive, psychotic symptoms. Other non-psychotic-symptom dominant TRS groups may, possibly, also be identified *IF* we had efficacious treatments for, e.g., negative symptoms, cognitive impairment, or social and vocational dysfunction. TRS infers resistance to dopamine D2 receptor (DAD2R) antagonism (through APD treatment) in relevant central nervous system (CNS) loci which may mediate symptomatic resistance. The specificity of resistance to D2 receptor antagonism to explaining TRS, though compelling, is tentative in view of clozapine, the only APD indicated to treat TRS, still does possess D2 receptor antagonism, though weak, as demonstrated by low *in vivo* human D2 striatal receptor occupancy [61%; (6)].

Are patients with TRS different from treatment responsive patients? Does this distinction reduce some of the heterogeneity in schizophrenia by “carving schizophrenia at a joint?” Unfortunately, much heterogeneity remains in TRS even after parsing it out from general schizophrenia. This persistent heterogeneity is based in part due to TRS patients demonstrating diversity in:

- Factors associated with poor response to treatment
- Onset of TRS in their disease course
- Response to clozapine (CLZ), the only approved treatment for TRS
- Inconsistent manner in which TRS has been defined across clinical research studies to date (4)
- Dominant symptom domains (e.g., positive, negative, cognitive) that are resistant to treatment.

Somewhat opposing views may consider TRS as a disease category distinct from general schizophrenia or perhaps rather as an outlier on a continuum of disease outcome severity, from full and adequate response and recovery to inadequate response to non-response, that characterizes schizophrenia (7). The continuum hypothesis posits that more severe pathophysiology leads to less response to treatment. Conversely, the categorical hypothesis presumes TRS patients suffer from a fundamentally different pathophysiology(s?) from that of the cohort with treatment responsive schizophrenia (8–10). The persistent challenge associated with both hypotheses is that the response/non-response dichotomy in either case is at best an arbitrarily defined boundary across dimensional measures of symptom severity.

A continuum of cumulative factors, or loading of factors, associated with poor response to treatment in schizophrenia may lead to TRS. Many genetic, developmental, behavioral, ethnocultural, and neurobiological factors have been associated with poor response or outcome in schizophrenia (11, 12) (Figure 2). TRS may be considered a consequence of diminished likelihood to respond favorably to treatment in the face of such overwhelming factors. Despite these associations, there are no clearly defined predictors of TRS nor even the likelihood to respond to a course of APD treatment.

**Figure 2:**
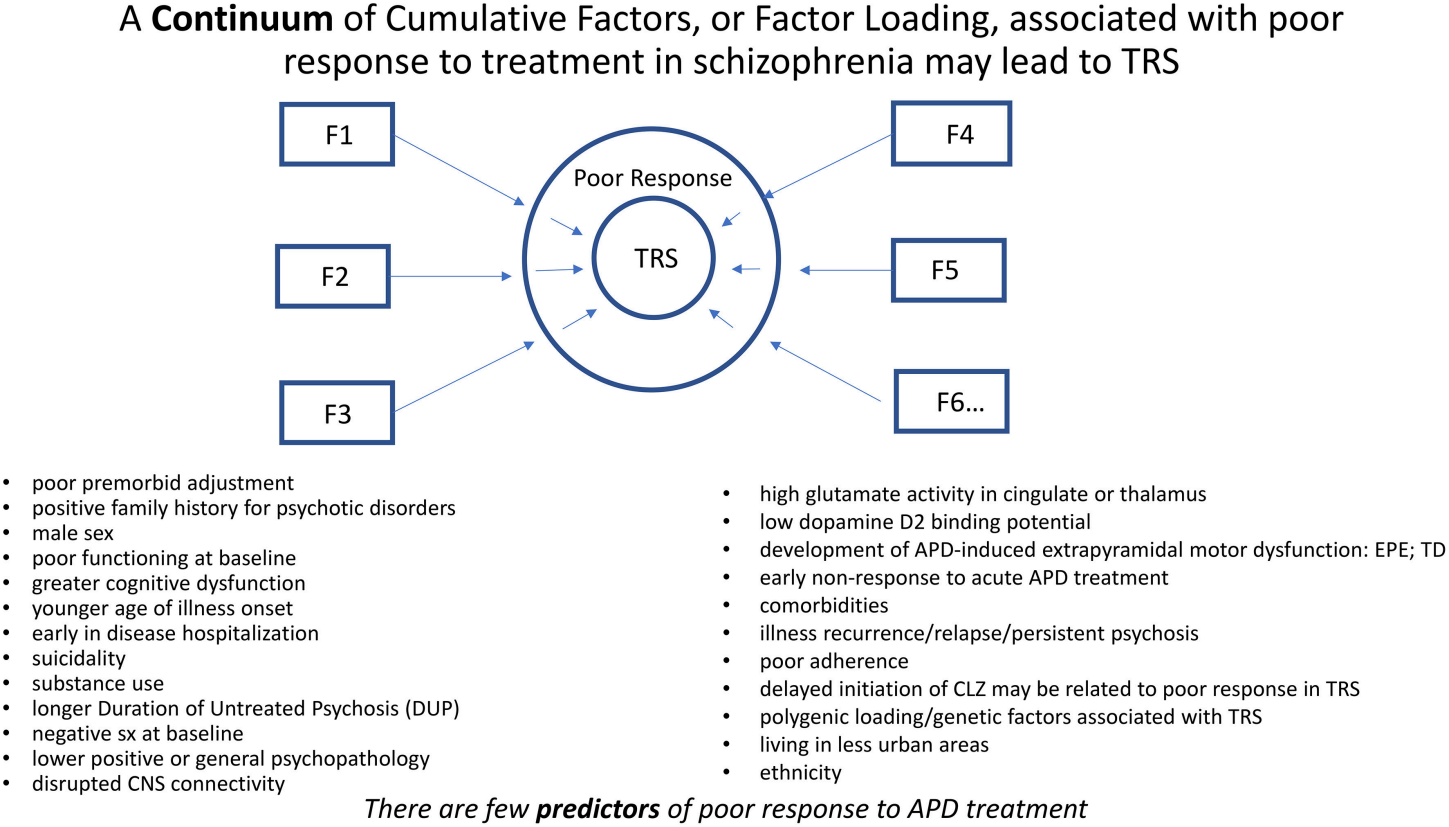
A continuum of cumulative factors (F*n*), as suggested by the above bulleted factors, may additively contribute (i.e., Factor Loading) to compromise response to treatment in schizophrenia. TRS may be considered a consequence of diminished likelihood to respond favorably to treatment in the face of such overwhelming factors. Despite these associations, there may be no clearly defined predictors of TRS.

Early non-response to acute APD treatment may predict subsequent non-response throughout the duration of that treatment episode. Of the few available predictors of APD response, early non-response suggests a plausible categorical distinction between TRS, and non-TRS patients. Early treatment responders, at 2 weeks, demonstrate better symptom improvement than early non-responders after a 12 week course of treatment with risperidone (13, 14). Early non-responders fail to achieve the same level of improvement seen in the early responders. The negative predictive value of early non-response has been reported extensively in the literature (15). Thus, the demonstration of early non-response to acute treatment may be a predictor for TRS as subsequent switching APDS has been shown to offer limited further efficacy for these early non-responders. The demonstration that switching treatment does not appear to be an effective treatment intervention in first episode patients failing their first course of APD treatment supports the observation that failure to respond is quite predictive of subsequent treatment failure (16, 17). Unfortunately, efforts to characterize who may be an early responder or non-responder prior to treatment trial and failure have not been very revealing.

The demonstration of inadequate response to APDs may infer that some TRS patients may be suffering from a non-dopamine pathophysiology since D2 receptor antagonist-based treatment is ineffective. Research has found through L-DOPA positron emission tomography (PET) imaging that patients with TRS may have “normal” rather than hyperactive dopamine synthesis and release in the striatum, whereas APD treatment responsive patients with schizophrenia do reveal significantly greater striatal dopamine activity compared to healthy controls. Conversely, patients with TRS seem to exhibit higher glutamate activity in the anterior cingulate based on glutamate magnetic resonance spectroscopy (MRS) imaging in contrast to treatment responsive patients (18). Therefore, dopamine D2 receptor antagonism may not have a significant influence on TRS symptoms. This provides initial support to consider TRS as a disease state categorically different from treatment responsive schizophrenia based on the apparent absence of a dopamine-based pathophysiology amenable to dopamine D2 receptor blockade. Further support for this neurobiological distinction awaits confirmation in other studies. TRS patients may additionally be distinguished from non-TRS patients by evidence of reduced brain gray matter volume (7, 19, 20), although this may not be a consistent finding across most studies in part due to the heterogeneity of the TRS population studied and inconsistencies in defining TRS (21). Other potentially distinguishing factors of TRS compared to non-TRS, such as gene profiling, polygenic loading, neurocognitive function, and demographics including non-urban residence (9, 10) also require further study and replication before any conclusions can be reached.

The categorical pathophysiologic distinction of TRS from that of general schizophrenia may be further illustrated in patients suffering from Primary TRS, or TRS occurring early in a patient's schizophrenia illness, within 5 years of illness onset (4, 22). Primary TRS is distinguished from Secondary TRS, or TRS occurring late in patient's illness (more than 5 years after illness onset) after a period of years of APD responsiveness. Up to 34% of all TRS may be Primary TRS (22). Many Primary TRS patients may never have demonstrated response to non-clozapine APDs or if so only briefly in the early course of their illness. Primary TRS may be associated with a normal- or hypo-dopaminergic CNS state. Few additional characteristics are presently known to distinguish Primary from Secondary TRS, other than possibly a higher proportion of males in Primary TRS (22).

Through utilization of an algorithm for the treatment of a first episode of schizophrenia, ~25% of patients were identified to be non-responders to either risperidone or olanzapine during their first treatment period, and of these, >80% again failed to respond to a subsequent second treatment trial when switched to the remaining treatment choice with either olanzapine or risperidone, respectively, (16). Therefore, this algorithm apparently identified Primary TRS patients whose failure to respond to non-clozapine APDs suggests that the symptoms of their first episode psychosis may not be mediated by an increase in dopaminergic activity nor at least improved by blocking the effects of a hyperdopaminergic state. Interestingly, after these 2 APD failures, the overwhelming majority of the non-responding patients (75%) when treated with clozapine now demonstrated an adequate treatment response, suggesting that clozapine may be mediating a treatment response through a mechanism beyond limited D2 receptor antagonism that may involve a non-dopamine pathophysiology. The limitations of this naturalistic algorithm-based study include no blinding of treatment, the patient's choice of first APD treatment received, and the relatively small number of patients who received clozapine (*n* = 28) in the third treatment trial compared to the number of patients who entered the first treatment trial (*n* = 244). An additional limitation might be that since both olanzapine and risperidone are mainly metabolized through CYP2D6, ultra-rapid metabolizers may have a reduced opportunity to respond to these APDs, whereas response to clozapine, in which CYP2D6 plays a minor metabolic role, may not be similarly disadvantaged. In a somewhat similar but larger and controlled switching clinical trial, (17) have recently reported that first episode patients who failed to achieve remission after an initial open-label trial on amisulpiride (44% non-remitters), later demonstrate a remission rate of <50% regardless of whether they subsequently receive double-blind treatment with either a switch to olanzapine or remaining on amisulpiride (56 and 55% non-remitters, respectively). A small number of these non-remitters went on to receive 12 week open-label clozapine treatment (*n* = 28); 5 of these patients (28%) remitted. These results further support the conclusion that first failure on a D2 antagonist APD may predict subsequent APD treatment failure in first episode schizophrenia patients. More data will be needed before one can conclude on the efficacy of clozapine in these first episode treatment resistant patients.

Therefore, risk factors associated with poor APD response in First Episode Schizophrenia (FES) should be associated with a hypo-dopaminergic state, or at least a “normo-” dopaminergic state, and these risk factors may similarly identify patients at risk for Primary TRS. Some risk factors that have been reported to be associated with poor response in FES include:

- Negative symptoms on illness presentation (23–25)
- Cognitive impairment at baseline (26) or during APD treatment (27)
- Continued substance abuse during early years in treatment (28, 29)
- Extrapyramidal side effects (EPS) during first APD treatment (26)
- Reduced DA activity, as evidenced by diminished frontal DA D2/3 binding potential as compared to APD responding FES patients (30).

These factors may be associated with a hypo-dopaminergic state and may therefore possibly reflect to some degree such state in treatment non-responsive FES patients. It is of course important to note that poor adherence to treatment may also be an overriding factor contributing to poor response in FES.

As first demonstrated in TRS by Demjaha et al. (18), a hyper-glutamatergic state has more specifically been associated with Primary TRS. FES patients with minimal APD exposure have been found to demonstrate an elevated glutamate MRS signal in the anterior cingulate as compared to healthy controls (31). This elevated glutamate signal is also seen in FES non-remitters as compared to remitters to APD treatment (32). These results provide further evidence to suggest that Primary TRS unlike treatment responsive schizophrenia may be a category of schizophrenia characterized more by a hyper-glutamatergic than a hyper-dopaminergic pathology. Of course, these neurotansmitters may be the result of proximal structural and/or genetic factors that may be primarily responsible for these distal distinctions between TRS and non-TRS patients.

Secondary TRS differs from Primary TRS in that the once APD responsive patient now no longer experiences an improvement in psychotic symptoms with APD treatment. This loss of response to APD treatment may conceivably be due to a progressive worsening of the underlying disease state or tolerance to the therapeutic effectiveness of continued DA D2 antagonism. First episode patients have been found to experience a progressive loss of APD response with each subsequent psychotic relapse experienced (33). This suggests that recurrent relapses may have a “neurotoxic” effect that worsens the underlying disease reducing the likelihood of full response to APD treatment (34, 35). Conversely, continuing treatment with APDs may have an iatrogenic effect, perhaps through chronic adaptive alteration of the dopamine receptor [e.g., pharmacologic tolerance due to dopamine receptor supersensitivity (36)] that over time contributes to Secondary TRS [i.e., Supersensitivity Psychosis (37)]. At present, no clear causative mechanism for Secondary TRS has been elucidated.

Clozapine (CLZ) stands as the only approved treatment for TRS. Unfortunately, not all TRS patients respond to an adequate treatment trial with CLZ (38). Thus, CLZ non-responsive as opposed to CLZ responsive patients further divide TRS into at least two additional patient segments. Possible factors associated with CLZ non-response (“Ultra-TRS”) include:

- Delayed initiation of CLZ (38–40)
- Cortical (temporal) thinning (41)
- Reduced glutamate activity (42)
- Polygenic factors (43, 44).

Ultra-TRS may reflect a schizophrenia disease state in which dopaminergic, glutamatergic, and perhaps much of the receptor pharmacology of available APDs have a greatly diminished influence on psychosis expression.

## Conclusions

A significant challenge to developing a new and effective treatment for TRS is the heterogeneity in the patient segments that make up what we refer to as *The Group of Treatment Resistant Schizophrenias*. By parsing these patient segments to achieve more homogeneous segments that may share a common pathophysiology, new drug development efforts for TRS may possibly emerge which may be more data driven, or at least hypothesis driven, than a “one size fits all” discovery strategy for a new treatment that may be efficacious in *all* TRS patients. A relevant first step may be to tentatively outline potential patient segments, in order from:

**A More Broadly Defined Segment…**

- All TRS patients?
- Fewer or greater load of poor response factors?
- Hypo-dopaminergic or hyper-glutamatergic activity?
- Early-in-Disease vs. Late-in-Disease?
- Fewer vs. greater number of failed treatment trials or relapses?
- Enhanced or diminished DAD2R signaling?
- History of response or non-response to clozapine?

**…to A More Targeted Segment**

At present, this concept has not been substantiated as targets that may mediate illness in specific patient segments have not been validated nor targeted therapies tested. Furthermore, parsing schizophrenia into patient segments based upon response to presently available treatments with all their limitations (e.g., little efficacy to improve such core symptoms as negative symptoms and cognitive impairment) may miss other, more fundamental neurobiological determinants of heterogeneity within schizophrenia. Lastly, consistency in defining TRS and diligence in ruling-out pseudo-TRS will be a requisite in all future studies in order to avoid clouding the pool of bonafide TRS cases.

As TRS remains an area of significant unmet medical need, a systematic effort to find new treatment alternatives must continue. Furthering our understanding of the basis for heterogeneity in TRS through the systematic investigation of relevant “clusters” of similarly at risk individuals for common neuropathology may hopefully bring us closer to realize a precision medicine approach from a clinical drug development strategy to target homogeneous TRS patient segments.

## Author Contributions

BK contributed to the intellectual content of the work including the conception and design of the work, the acquisition and interpretation of data and literature for the work, the critical drafting and review of the work, and the final approval for this work to be published.

### Conflict of Interest Statement

BK is an employee of Lundbeck North America, a pharmaceutical company that develops drugs for CNS disorders. This manuscript though was developed completely by the author BK who has expressly stated in the work that the opinions expressed are solely those of the author and not necessarily those of Lundbeck.
